# Comparative Genomics and Transcriptomics of the Extreme Halophyte *Puccinellia tenuiflora* Provides Insights Into Salinity Tolerance Differentiation Between Halophytes and Glycophytes

**DOI:** 10.3389/fpls.2021.649001

**Published:** 2021-04-22

**Authors:** Rui Guo, Long Zhao, Kaijian Zhang, Huiying Lu, Nadeem Bhanbhro, Chunwu Yang

**Affiliations:** ^1^Key Laboratory of Dryland Agriculture, Institute of Environment and Sustainable Development in Agriculture, Chinese Academy of Agricultural Sciences, Beijing, China; ^2^Key Laboratory of Molecular Epigenetics of Ministry of Education (MOE), Northeast Normal University, Changchun, China; ^3^Institute of Genetics and Developmental Biology, Chinese Academy of Sciences, Beijing, China; ^4^Beijing Novogene Bioinformatics Technology Ltd., Beijing, China

**Keywords:** gene family expansion, genome, halophyte, *Puccinellia tenuiflora*, RNA-seq, positive selection

## Abstract

Halophytes and glycophytes exhibit clear differences in their tolerance to high levels of salinity. The genetic mechanisms underlying this differentiation, however, remain unclear. To unveil these mechanisms, we surveyed the evolution of salinity-tolerant gene families through comparative genomic analyses between the model halophyte *Puccinellia tenuiflora* and glycophytic Gramineae plants, and compared their transcriptional and physiological responses to salinity stress. Under salinity stress, the K^+^ concentration in the root was slightly enhanced in *P. tenuiflora*, but it was greatly reduced in the glycophytic Gramineae plants, which provided a physiological explanation for differences in salinity tolerance between *P. tenuiflora* and these glycophytes. Interestingly, several K^+^ uptake gene families from *P. tenuiflora* experienced family expansion and positive selection during evolutionary history. This gene family expansion and the elevated expression of K^+^ uptake genes accelerated K^+^ accumulation and decreased Na^+^ toxicity in *P. tenuiflora* roots under salinity stress. Positively selected *P. tenuiflora* K^+^ uptake genes may have evolved new functions that contributed to development of *P. tenuiflora* salinity tolerance. In addition, the expansion of the gene families involved in pentose phosphate pathway, sucrose biosynthesis, and flavonoid biosynthesis assisted the adaptation of *P. tenuiflora* to survival under high salinity conditions.

## Highlights

- The evolution of salinity-tolerance genes has contributed to the development of salinity tolerance in the extreme halophyte *Puccinellia tenuiflora*.
- Root potassium concentration was slightly enhanced in *P. tenuiflora* under salinity stress, but it was greatly reduced in glycophytic Gramineae plants.
- Several potassium channel and potassium transporter gene families from *P. tenuiflora* were expanded and positively selected during the species’ evolutionary history.

## Introduction

Soil salinization is a severe environmental problem that limits agricultural production (Flowers and Yeo, 1995; Flowers et al., 2010). Saline soil contains various concentrations and types of salts, and soil salinity levels can reach 2,380 mM Na^+^. Salt-affected soils may contain NaCl alone, NaCl and Na_2_SO_4_, or NaCl and Na_2_CO_3_. Plant growth under saline conditions is influenced by multiple stress factors, including direct ion injury and osmotic stress inflicted by Na^+^ and Cl^–^ and indirect nutrient stresses caused by competition from Na^+^ and Cl^–^ (Munns and Tester, 2008). Tolerance to salinity stress is imparted by a complex network that affects almost all plant metabolic and developmental processes that are controlled by numerous genes. Higher plants can be classified as halophytes or glycophytes according to their ability to tolerate salinity stress. Halophytes can survive and spend their entire life cycle at ≥200 mM NaCl (Flowers et al., 2010). Many halophytes can even survive conditions of >1000 mM salinity, whereas most glycophytes can only survive under conditions of 50–100 mM salinity (Munns and Tester, 2008). The molecular study of salinity tolerance has largely focused on model glycophytes, such as rice and Arabidopsis (Munns and Tester, 2008; Suzuki et al., 2016; Wang and Xia, 2018; Ganie et al., 2019; Wang et al., 2019; Zhu et al., 2019), however, relatively few attentions have been paid to the molecular mechanisms underlying salinity tolerance in halophytes.

Although halophytes and glycophytes exhibit dramatic differences in salinity tolerance, they share most of the characteristics necessary for survival in saline soils. These include the control or excretion of Na^+^ and Cl^–^ in the root, compartmentalization of toxic ions in organs or cells, synthesis of compatible organic solutes in the cytoplasm, and maintenance of sufficient concentrations of key nutrients such as K and N (Flowers and Colmer, 2008; Wang and Xia, 2018). Halophytes have evolved a strong tolerance to salinity over history and the role played by genome evolution in establishing differences in salinity tolerance between halophytes and glycophytes is of considerable interest.

*Puccinellia tenuiflora* (2*n* = 14) is a forage grass with high nutritional value and strong tolerance for salinity and alkaline stresses (Yan and Sun, 2000; Zhao et al., 2011; Meng et al., 2016). This species is even able to grow normally under extreme conditions (e.g., salinity >600 mM and pH 10–11) (Xu, 1990; Xu et al., 1995; Wang et al., 2006; Zhang et al., 2013). Many molecular studies of salinity-tolerance have focused on this halophyte (Wang et al., 2007a, b; Liu et al., 2009; Ardie et al., 2009, 2010, 2011; Yu et al., 2011; Zhang et al., 2013, 2017; Yu et al., 2013). To reveal the regulation of gene expression and genomic mechanisms underlying the differences in salinity tolerance between halophytes and glycophytes, we conducted comparative genomic and transcriptome analyses between *P. tenuiflora* and several glycophytic Gramineae plants. Our results improved the current understanding of salinity tolerance in halophytes and provided insights into the mechanisms underlying differences between halophytes and glycophytes.

## Materials and Methods

### Plant Materials and Reference Genome

The taxonomy characteristics of *Puccinellia tenuiflora* are available at Flora of China^1^). *P. tenuiflora* was bred as a grass variety and is not an endangered or protected species, so no specific permissions were required for its application in this study. The reference genome of *P. tenuiflora* used in this study was reported in our previous publication (Guo et al., 2020). The genome of *Dactylis glomerata* was reported in Huang et al. (2020). The reference genome with integrated annotation files of *D. glomerata* is publicly available at https://orchardgrassgenome.sicau.edu.cn/. In the current study, we used the *P. tenuiflora* variety ‘Baicheng’ for RNA sequencing (RNAseq) and physiological measurements. The *P. tenuiflora* seeds were provided by Jilin Academy of Agricultural Sciences, Changchun, China.

### Gene Family Discovery

Gene families were discovered using OrthoMCL software^2^ (Li et al., 2003). The whole genome sequences, protein sequences, and transcript sequences of 16 species (*Thellungiella salsuginea*, *Arabidopsis thaliana*, *Phalaenopsis equestris*, *Elaeis guineensis*, *D. glomerata, Musa acuminata*, *Oryza sativa*, *Brachypodium distachyon*, *Setaria italica*, *Zea mays*, *Hordeum vulgare*, *Aegilops tauschii*, *Sorghum bicolor*, *Ananas comosus*, *Zostera marina*, and *Amborella trichopoda*) were obtained from NCBI, Ensembl, and http://marinegenomics.oist.jp/gallery/. To exclude putative fragmented genes, the genes encoding protein sequences shorter than 50 amino acids were filtered (Yuan et al., 2018). The longest transcript was selected if a gene showed alternative splicing. Finally, gene family clustering was carried out using OrthoMCL software with a 1.5 inflation index (Li et al., 2003). We used InterProscan software and the Pfam motifs of Late Embryogenesis Abundant (LEA)1, LEA2, LEA3, LEA4, LEA5, DEHYDRIN, and Seed Maturation Protein (SMP) to discover the LEA superfamily members present in the genomes of *P. tenuiflora*, *O. sativa*, *H. vulgare, A. tauschii, B. distachyon*, and *D. glomerata* (Finn et al., 2017). We constructed the polygenetic tree of the *LEA5* family using the MEGA-X-based Neighbor-Joining method (Kumar et al., 2018).

### Phylogenetic Tree and Divergence Time

We applied the sequences of 228 single copy orthologs to generate the phylogenetic tree. We aligned the protein sequences of these orthologs using MUSCLE software (Edgar, 2004), and then the protein alignments were translated to CDS sequences. The phylogenetic tree was generated using RAxML (for maximum likelihood; 7.2.3) (Stamatakis, 2006; Stamatakis et al., 2008). The MCMCTREE model of PAML was selected to calculate the divergence time between *P. tenuiflora* and the other 16 species (main parameters: burn-in = 10,000, sample-number = 100,000, sample-frequency = 2, and clock = 2) (Yang, 2007). Three calibration points used were based on the study by Cai et al. (2015) and the TimeTree website^3^.

### Expansion and Contraction of Gene Families and Positive Selection Analysis

Genes from different species were clustered into gene families using OrthoMCL software (see Text Footnote 3) with a 1.5 inflation index (Li et al., 2003). The gene families were excluded from the analysis if the gene number was ≥200 in one species or ≤2 in all other species. The Cafe software package was applied to determine the expansion and contraction of gene families (De Bie et al., 2006; Han et al., 2013). Gene gains and losses in gene families in a phylogeny were tested by calculating the *P*-values of each branch using the Viterbi method with a randomly generated likelihood distribution. The exact *P*-values for transitions between the parent and child family sizes were estimated using this method for all branches of the phylogenetic tree (De Bie et al., 2006; Han et al., 2013). Positive selection analysis was based on the alignments of protein sequences. We used MUSCLE software to align the protein sequences of single copy genes in *P. tenuiflora*, *O. sativa*, *H. vulgare*, and *A. tauschii* (Edgar, 2004) and Gblocks to filter poor positions and transform protein alignments to CDS (Castresana, 2000). Positive selection sites were discovered with the branch-site model (Zhang et al., 2005) of PAML (Yang, 2007) by setting *P. tenuiflora* as the foreground branch. The *P*-values were generated using the χ^2^ test, and then adjusted by the false discovery rate (FDR) method.

### LTR Retrotransposons Insert Time Assessment

Nucleotide variations at the 5′ and 3′ ends of intact LTR retrotransposons and DNA substitution rates were calculated (Huang et al., 2020). The nucleotide variations and DNA substitution rates were used to estimate the insert time of LTR retrotransposons according to the method described by Ma and Bennet-zen (2004).

### Motif Analysis

We carried out a motif analysis of the low affinity K^+^ transporter (AKT), Na^+^/H^+^ exchanger (NHX), and high-affinity K^+^ transporter (HKT) proteins among *O. sativa*, *H. vulgare*, *A. tauschii*, *P. tenuiflora*, and *D. glomerata* using MEME Suite 5.2 (Bailey et al., 2009). The orthologous genes of *AKT*, *HKT*, and *NHX* for each species were identified by the best BLASTn hit against the corresponding rice sequence (qcovs ≥0.5, *E*-value ≤−7, and identity ≥60%).

### Comparative Transcriptome and Physiological Measurement of *P. tenuiflora* and Glycophytic Gramineae Plants

*Seeds* of *P. tenuiflora* (variety Baicheng), *O. sativa* (variety JIJING88), *H. vulgare* (variety Zhu4300), *D. glomerata* (variety Sparta), and *Triticum aestivum* (variety Chinese Spring) were planted in plastic pots containing washed sand under greenhouse conditions (24–26°C day and 18–21°C night under 16-h light). The rice and wheat seeds were provided by Dr. Bao Liu (Northeast Normal University, Changchun, China), and the *H. vulgare* seeds and *D. glomerata* seeds were provided by the Chinese Academy of Agricultural Sciences (Beijing, China). The pots (15 plants per pot) were cultured for 15 days with half-strength Hoagland nutrient solution. We stressed 15-day-old seedlings (at about the three leaves stage) through daily exposure to half-strength Hoagland nutrient solution containing 300 mM NaCl. Control plants were watered with half-strength Hoagland nutrient solution. We used a randomized complete block design. After 12 and 48 h of exposure to salinity stress, we collected physiological measurement samples from all of the five species and RNA samples from *P. tenuiflora* and *D. glomerata*. There were three biological replicates of each treatment and tissue, and each biological replicate comprised a pool of 5–20 plants. A total of 2 μg RNA per sample was used as the input material for the RNA sample preparations. Sequencing libraries were generated using a NEBNext UltraTM RNA Library Prep Kit for Illumina (NEB, United States) following manufacturer’s recommendations and index codes were added to attribute sequences to each sample. All downstream analyses were performed with high quality clean data. About 6 Gb of clean data for each sample were used to perform the transcriptional analysis. Statistically significant differentially expressed genes (DEGs) were discovered using the DESeq2 R package, and DEG was defined by the adjusted *P*-value (FDR) <0.05 and Fold Change ≥2. The DEGs were subjected to gene ontology (GO) and Kyoto Encyclopedia of Genes and Genomes (KEGG) enrichment analyses with a GOseq R package (Young et al., 2010) and KOBAS software (Mao et al., 2005), respectively. We discovered ortholog of each *P. tenuiflora* gene in the *D. glomerata* genome using the best BLAST hit based on the CDS sequence (bit score ≥200, *E*–value ≤−7, and identified ≥60%). Quantitative real-time PCR (qRT-PCR) was employed to validate the results of the transcriptome analysis. RNA samples from the *P. tenuiflora* roots taken at 48 h after the onset of salt treatment were treated with DNaseI (Invitrogen), reverse-transcribed using SuperScript^TM^ RNase HReverse Transcriptase (Invitrogen), and then subjected to qRT-PCR analysis. Amplification of the target genes was monitored by SYBR Green. *Actin* and *GAPDH* were used as internal control genes. The gene expression data were analyzed using the ΔΔ*C*t method (Livak and Schmittgen, 2001). Dried samples of roots and shoots were digested in 65% HNO_3_ at 120°C, and their Na^+^ and K^+^ contents were measured using an atomic absorption spectrophotometer (TAS-990super, PERSEE, China). The statistical analysis (*t*-test) of the qRT-PCR and physiological measurements was performed using SPSS 16.0 (SPSS, Chicago, IL, United States).

## Results

### Gene Family Analysis

**FIGURE 1:**
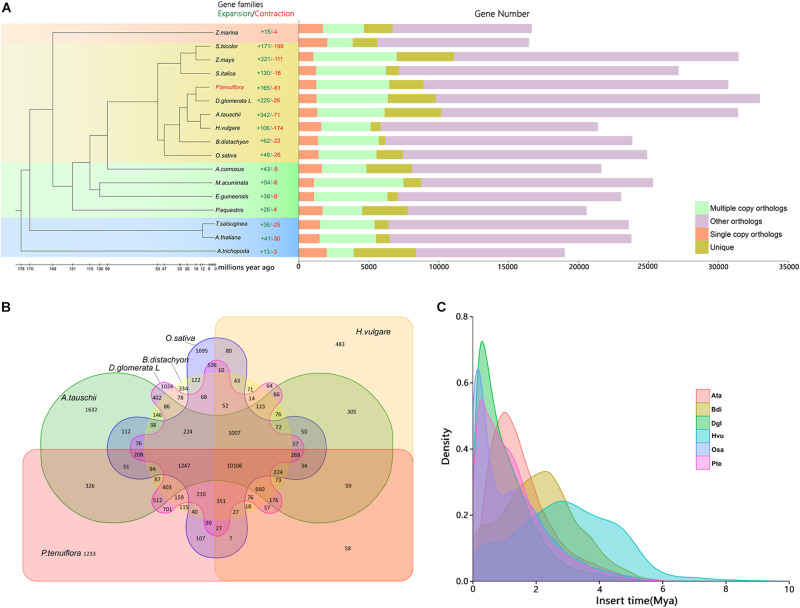
Gene family, phylogenetics, and time of LTR-retrotransposon insertion in *Puccinellia tenuiflora.* **(A)** Phylogenetic relationship of *P. tenuiflora* and 16 plant species with sequenced genomes. The phylogenetic tree was constructed using maximum-likelihood analysis and alignment of 228 single copy proteins. **(B)** Venn diagram of gene family comparison among *P. tenuiflora* and several sequenced Gramineae plants. **(C)** Time of LTR-retrotransposon insertion in *P. tenuiflora* (Pte), *Oryza sativa* (Osa), *Dactylis glomerata* (Dgl), *Hordeum vulgare* (Hvu), *Aegilops tauschii* (Ata) and *Brachypodium distachyon* (Bdi).

**FIGURE 2:**
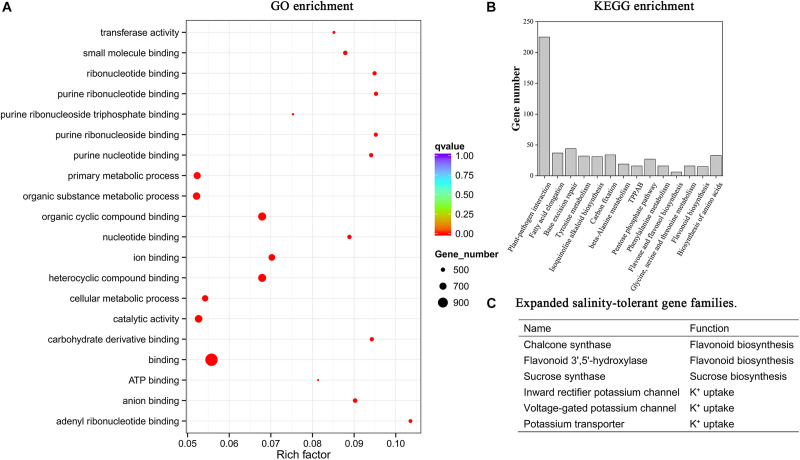
Gene family expansion analysis of *P. tenuiflora* during evolutionary history. Gene family expansion analysis was conducted using OrthoMCL and Cafe software. **(A)** GO enrichment of *P. tenuiflora* gene families that expanded during evolutionary history. Only the top 20 enriched GO terms are displayed. **(B)** KEGG enrichment of the *P. tenuiflora* gene families that expanded during evolutionary history. Only the enriched KEGG pathways with adjusted *P*-values < 0.05 are displayed. **(C)** Expanded salinity-tolerant gene families of *P. tenuiflora* during evolutionary history.

The point of divergence between *P. tenuiflora* and *D. glomerata* was about 12.8 million years ago (Figure 1A). A total of 1, 233 gene families were unique to *P. tenuiflora* and potentially related to the strong salinity tolerance found in this species (Figure 1B). The gene number of *P. tenuiflora* was similar to that of *O. sativa*, *Z. mays*, *D. glomerata*, and *S. italica* (Supplementary Figure 1). The genome of *P. tenuiflora* contained 691 Mb of transposable elements (62.44% of the total sequence), including 580 Mb of LTR retrotransposons (52.43%) (Guo et al., 2020). We estimated the insertion time for the LTRs in *P. tenuiflora, O. sativa, D. glomerata, H. vulgare, A. tauschii*, and *B. distachyon* (Figure 1C). The LTR insertion peak indicates that a frequent insertion event occurred. The LTR insertion peak of *P. tenuiflora* was between those of *O. sativa* and *D. glomerata* (Figure 1C). The frequent LTR insertion events for *P. tenuiflora* occurred much later than those of its close relative, *H. vulgare*. We applied OrthoMCL software and Cafe software to conduct the expansion and contraction analysis of the gene family and members of all of the expanded *P. tenuiflora* gene families are listed in Supplementary Table 1. We conducted GO and KEGG enrichment analyses for all of the expanded genes listed in Supplementary Table 1. The GO enrichment analysis indicated that the expanded *P. tenuiflora* genes were enriched in many metabolic processes such as ion binding, sucrose synthase, G-protein activated inward rectifier potassium channel activity, organic substance metabolic process, and primary metabolic process (Supplementary Table 2 and Figure 2A). The KEGG enrichment showed that the expanded *P. tenuiflora* genes were enriched in the plant-pathogen interaction pathway, pentose phosphate pathway (PPP), phenylalanine metabolism, flavone and flavonol biosynthesis, flavonoid biosynthesis, and biosynthesis of amino acids (Figure 2B). We were particularly interested in expanded salinity-tolerance gene families and found that the inward rectifier potassium channel, voltage-gated potassium channel, potassium transporter, flavonoid 3′,5′-hydroxylase, chalcone synthase, and sucrose synthase gene families of *P. tenuiflora* were expanded (Figure 2C and Supplementary Table 1).

**FIGURE 3:**
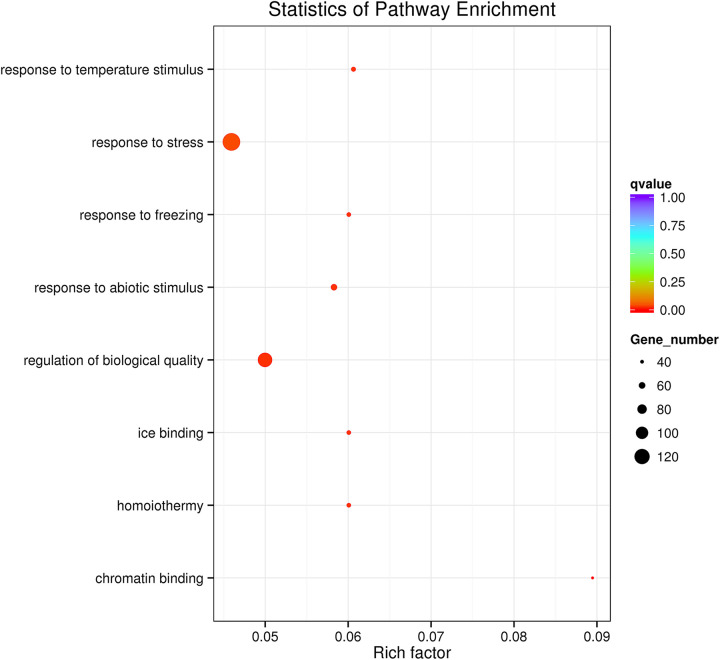
Gene ontology enrichment of *P. tenuiflora* genes that were positively selected during evolutionary history.

Positive selection analysis revealed that 1,324 *P. tenuiflora* genes were positively selected over time (Supplementary Table 3). These genes were enriched in only eight GO terms, including the response to abiotic stimulus, response to temperature stimulus, response to freezing, and response to stress (Figure 3). Many salinity-tolerant genes of *P. tenuiflora* were found also to have been positively selected during evolutionary history, including six voltage-gated potassium channel genes, two inward rectifier potassium channel genes, and one potassium channel gene (Table 1).

**TABLE 1:** *Puccinellia tenuiflora* salinity-tolerant genes that were positively selected during evolutionary history.

Gene ID	Function description
PtO_1679.40	Inward rectifier potassium channel Kir2.1
PtO_1088.1	Inward rectifier potassium channel Kir2.2
PtF_293.37	Potassium channel THIK
PtO_1952.35	Voltage-gated potassium channel
PtF_523.125	Voltage-gated potassium channel Kv1.3
PtO_1553.15	Voltage-gated potassium channel Kv3.4
PtO_1172.14	Voltage-gated potassium channel Kv4.3
PtF_19.3	Voltage-gated potassium channel KCNQ1
PtO_1180.6	Voltage-gated potassium channel Kv1.6

### Evolutionary History of Salinity-Tolerance Families

**FIGURE 4:**
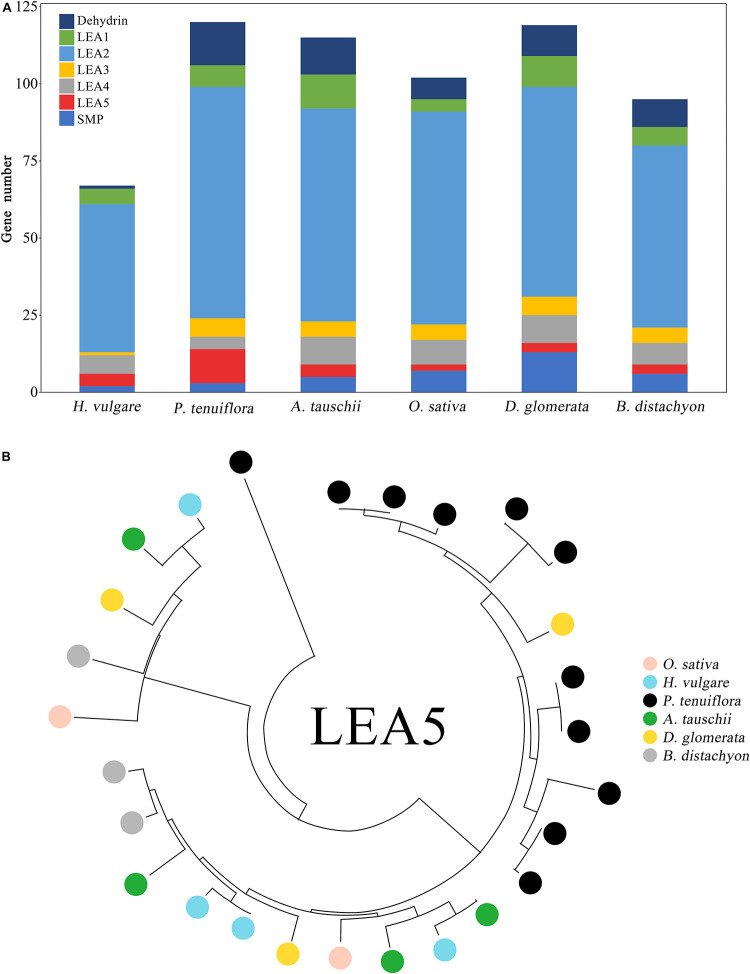
LEA superfamily of *P. tenuiflora*. **(A)** Comparison of the member numbers of the *LEA* superfamily in *P. tenuiflora*, *D. glomerata*, rice, *H. vulgare*, *B. distachyon*, and *A. tauschii*. **(B)** Polygenetic tree of the *LEA5* families in *P. tenuiflora*, *D. glomerata*, rice, *H. vulgare*, *B. distachyon*, and *A. tauschii*. Polygenetic tree constructed by MEGA-X using the Neighbor-Joining method.

Here we focused on the evolution of several critical salinity tolerance gene families including the *HKT*, *AKT*, *NHX*, and *LEA* families (Figure 4 and Supplementary Figures 2–4). The *LEA* superfamily is divided into seven subfamilies on the basis of their distinctive motifs: *LEA1*, *LEA2*, *LEA3*, *LEA4*, *LEA5*, *SMP*, and *DEHYDRIN*. The member number in the *LEA5* subfamily of *P. tenuiflora* was much higher than that of *D. glomerata*, *O. sativa*, *H. vulgare*, *B. distachyon*, and *A. tauschii* (Figure 4A). Although the gene number in *P. tenuiflora* was much lower than that in *H. vulgare* and *A. tauschii* (Supplementary Figure 1), the *LEA5* number was much higher in *P. tenuiflora* than in *H. vulgare* and *A. tauschii* (Figure 4A). We generated a phylogenetic tree of the *LEA5* families of *P. tenuiflora*, *O. sativa*, *H. vulgare*, *B. distachyon*, *A. tauschii*, and *D. glomerata*. The phylogenetic tree illustrated that the *LEA5* family of *P. tenuiflora* expanded via duplication (Figure 4B). We conducted a comparative protein motif analysis of *P. tenuiflora* and several glycophytic Gramineae plants for *AKT, HKT*, and *NHX* genes and found that *P. tenuiflora* and the glycophytic Gramineae plants displayed similar protein motif components for all of the selected genes (Supplementary Figures 2–4).

### Ion Content

**FIGURE 5:**
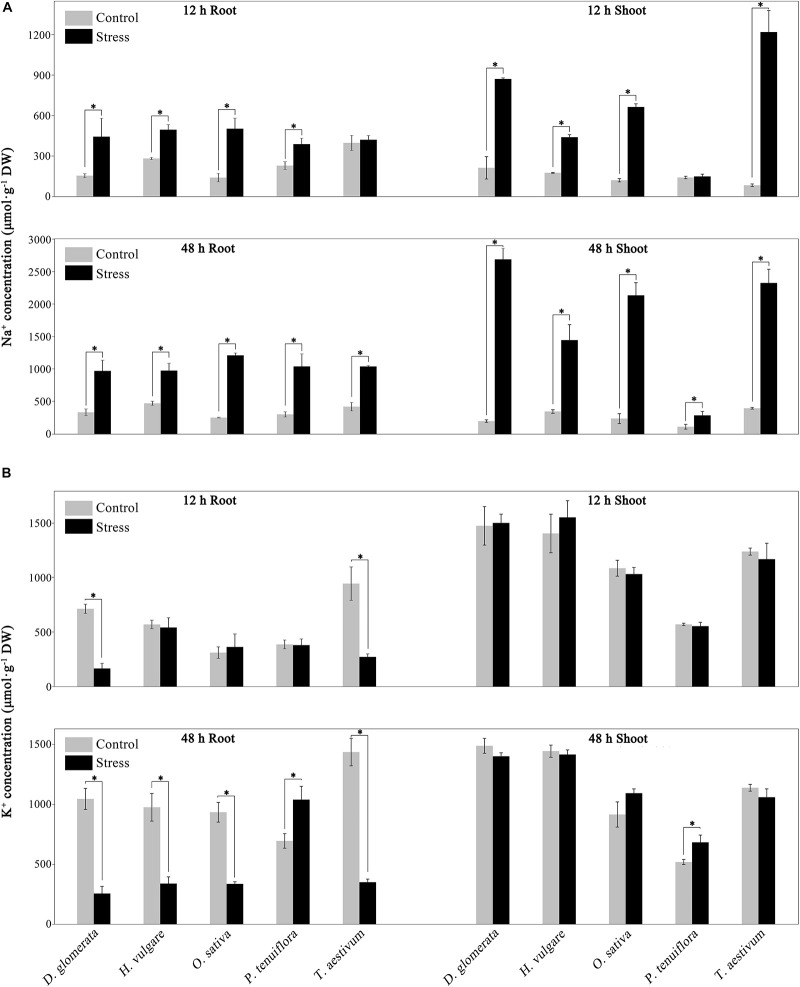
Effects of salinity stress on Na^+^ **(A)** and K^+^ **(B)** concentration in *P. tenuiflora*, *O. sativa*, *H. vulgare*, *D. glomerata*, and *T. aestivum*. The 15-day-old seedlings were exposed to 300 mM NaCl for 12 and 48 h. Values are expressed as means (±standard deviation, S.D.) of the three biological replicates. ^∗^Indicates a significant difference between control and stress conditions within the same tissue at the 0.05 level, according to the *t*-test.

In the shoots of all of the four glycophytic Gramineae plants, the Na^+^ concentration was greatly enhanced under salinity stress at both the 12 and 48 h time points (Figure 5A). However, the shoot Na^+^ concentration in *P. tenuiflora* was unaffected at 12 h and slightly enhanced at 48 h under salinity stress (Figure 5A). Under salinity stress, *P. tenuiflora* and the glycophytic Gramineae plants displayed similar levels of Na^+^ in their roots (Figure 5A). The K^+^ accumulation in the shoots was unaffected in all of the four glycophytic Gramineae plants at both 12 and 48 h but enhanced in *P. tenuiflora* at 48 h (Figure 5B). When the seedlings were exposed to salinity stress for 12 h, the root K^+^ concentration decreased in *D. glomerata* and *T. aestivum* but not in *H. vulgare*, *O. sativa*, or *P. tenuiflora*. However, when the seedlings were exposed to salinity stress for 48 h, the concentration of K^+^ in the roots significantly decreased in all of the four glycophytic Gramineae plants, while being elevated by 49.5% in *P. tenuiflora* (Figure 5B). Under salinity stress, the Na^+^/K^+^ ratio was much lower in *P. tenuiflora* than in the four glycophytic Gramineae plants in both shoots and roots at 48 h (Supplementary Figure 5).

### Transcriptional Profiling of *P. tenuiflora* Under Salinity Stress

When the seedlings were exposed to salinity stress for 12 h, 1,628 DEGs were present in the roots and 760 in the leaves of *P. tenuiflora*. When the seedlings were exposed to salinity stress for 48 h, 2,068 DEGs were present in the roots and 1,109 in the leaves of *P. tenuiflora* (Supplementary Table 4). We subjected the DEGs to KEGG enrichment analyses (Supplementary Figure 6), specifically focusing on the DEGs involved in K^+^/Na^+^ homeostasis and osmotic adjustment (Supplementary Table 5). In the roots of *P. tenuiflora*, a *HAK* (KUP/HAK/KT K^+^ transporter) gene (ID: PtO_2103.26) and *HKT1:3* gene (ID: PtO_1129.21) were upregulated at 48 h, and two *voltage-gated potassium channel* genes (IDs: PtO_1129.2 and PtF_428.24) were upregulated at both 12 and 48 h (Supplementary Table 5). Two *NHX* (Na^+^/H^+^ exchanger) genes and a *V-H*^+^*- ATPase* gene were upregulated in the roots of *P. tenuiflora* at 12 h or 48 h but not in the leaves (Supplementary Table 5). Twenty-nine *LEA* genes and 12 *dehydrin* genes were upregulated in the roots or leaves of *P. tenuiflora* (Supplementary Table 5). *Glucose-6-phosphate dehydrogenase* (*G6PDH*) is the rate-controlling gene for the PPP, *sucrose synthase* genes are central to sucrose biosynthesis, and *flavonoid 3*′,*5*′*-hydroxylase* genes are critical to flavonoid biosynthesis. Two *flavonoid 3*′,*5*′*-hydroxylase* genes (IDs: PtF_517.39 and PtO_820.20) were upregulated in the leaves of *P. tenuiflora* at 12 h, and *G6PDH* (PtO_647.1) was upregulated in the roots of *P. tenuiflora* at 12 h (Supplementary Table 5). Three *sucrose synthase* genes (IDs: PtO_1407.1, PtO_1927.24, and PtO_999.27) were upregulated in the roots or leaves of *P. tenuiflora*. Five *abscisic acid (ABA) responsive element binding factor* (*ABF*) genes, which are core ABA signaling genes and two *9-cis-epoxycarotenoid dioxygenase* (*NCED*) genes, which are critical to ABA biosynthesis, were upregulated in the roots or leaves of *P. tenuiflora*. Some *superoxide dismutase* genes and *glutathione S-transferase* genes were also upregulated in *P. tenuiflora*. The qRT-PCR experiment was carried out using gene-specific primers to validate the results of the RNAseq experiment (Supplementary Table 6). For eight of the 11 genes tested, the fold-change values from the RNAseq were similar to those from the qRT-PCR data, indicating that the results from the RNAseq were reliable (Supplementary Table 6).

### Comparative Orthologous Expression Response of *P. tenuiflora* and *D. glomerata*

**FIGURE 6:**
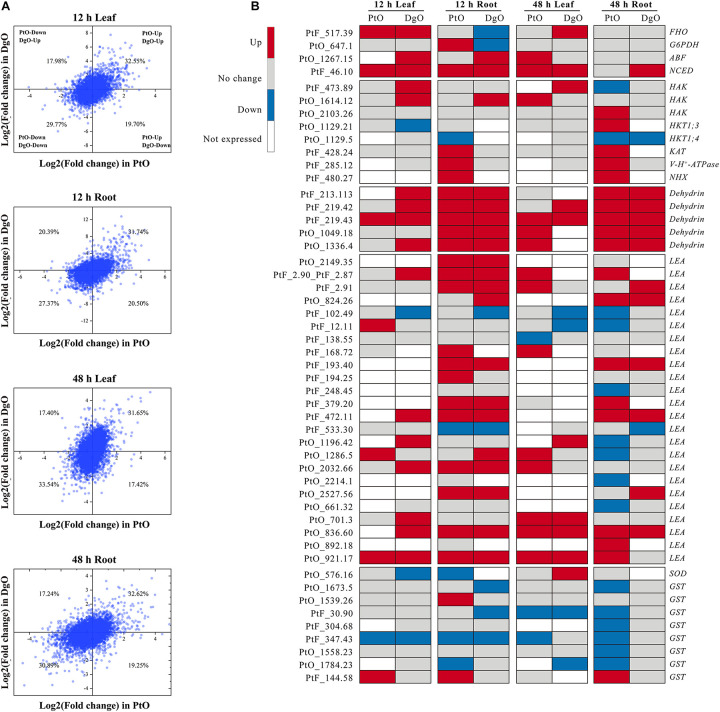
Comparative orthologous expression response of *P. tenuiflora* and *D. glomerata* to salinity stress. **(A)** Rectangular coordinate system with *x* = log_2_ [fold change (stress/control) of PtO] and *y* = log_2_(fold change of DgO) for general genes. **(B)** Heat map showing the expression response of the *P. tenuiflora* ortholog (PtO) and *D. glomerata* ortholog (DgO) to salinity stress for each salinity tolerance gene. The 15-day-old seedlings were exposed to 300 mM NaCl for 12 and 48 h. Each treatment had three biological replicates. FHO, flavonoid 3′,5′-hydroxylase; G6PDH, glucose-6-phosphate dehydrogenase; ABF, ABA responsive element binding protein; NCED, 9-*cis*-epoxycarotenoid dioxygenase; HAK, KUP/HAK/KT K^+^ transporter; HKT, high-affinity K^+^ transporter; KAT, potassium channel KAT; LEA, late embryogenesis abundant protein; NHX, Na^+^/H^+^ exchanger; SOD, superoxide dismutase; GST, glutathione *S*-transferase.

To compare the gene expression response of *P. tenuiflora* and *D. glomerata* to salinity stress, we identified the ortholog of each *P. tenuiflora* gene in the *D. glomerata* genome using the BLAST program. We displayed the gene expression difference between *P. tenuiflora* and *D. glomerata* in a rectangular coordinate system with *x* = log2 (fold change of the *P. tenuiflora* ortholog) and *y* = log2 (fold change of the *D. glomerata* ortholog) for each gene; the fold change was the ratio of stress to control of the expression level (Figure 6A). About 60% of the expressed genes were present in quadrant I and quadrant III, in which the *P. tenuiflora* ortholog (PtO) and *D. glomerata* ortholog (DgO) displayed similar responses to salinity stress (Figure 6A). We also compared the expression response of the PtO and DgO to salinity stress for each salinity-tolerance gene (Figure 6B). For several of the *LEA* genes, the PtO and DgO were upregulated either in the roots or leaves. Both the PtO and DgO showed upregulation of five *dehydrin* genes in roots at both 12 and 48 h. The PtO and DgO of a *flavonoid 3*′,*5*′*-hydroxylase* gene were upregulated in leaves at 12 h. *G6PDH* was upregulated in roots of *P. tenuiflora* but not in roots of *D. glomerata* at 12 h. Both the PtO and DgO of *NCED* gene were upregulated in leaves at 12 and 48 h and in roots at 12 h (Figure 6B). The PtO of the *ABF* gene was upregulated in leaves at 48 h and that of the DgO was upregulated in both leaves and roots at 12 h (Figure 6B). *HKT1;3* was upregulated in the roots of *P. tenuiflora* but not in the roots of *D. glomerata* at 48 h (Figure 6B). The *potassium channel KAT* gene (*KAT*), *V-H*^+^*-ATPase* gene, and *NHX* gene were upregulated in the roots of *P. tenuiflora* at both 12 and 48 h but not in roots of *D. glomerata*. In summary, PtO and DgO exhibit similar expression responses to salinity stress in the ABA pathway, *dehydrin*, and *LEA* genes, but they displayed different expression responses in *HKT1;3*, *V-H*^+^*-ATPase*, *NHX*, and *G6PDH*.

## Discussion

### Comparative Genomics

The comparative genomics results showed that the frequent LTR insertion event of *P. tenuiflora* occurred later than that of its close species, *H. vulgare* and *D. glomerata*. The LTR insertion events in *P. tenuiflora* occurred mostly during the Pleistocene Epoch, which lasted from approximately 2.6 million years to 11,700 years ago, including at least five documented major ice ages. Repeated ice ages (glacial cycles) can result in frequent change in sea-level due to the exchange of water between ice and ocean (Lambeck et al., 2014). The circular change of sea level may increase land salinity, which may be the main factor for the formation of extant saline land. During this period, the LTR of *P. tenuiflora* was frequently activated, which led to the rapid reorganization of its genomes to adapt to increasing soil salinity. *P. tenuiflora* also has very strong resistance to chilling stress (Zhao et al., 2011, 2016). The frequent LTR insertion events in *P. tenuiflora* during the Pleistocene Epoch were likely to have contributed to the development of its strong tolerance to cold and multiple other stresses. In addition, *P. tenuiflora* genes positively selected during evolution were enriched in response to temperature stimulus, response to freezing, and response to stress (Figure 3), revealing that the evolution of functional genes may also have contributed to the development of tolerance to multiple stress types, including salinity stress and chilling stress.

### ABA Signaling and Osmotic Adjustment Mechanisms Under Salinity Stress May Be Conserved in *P. tenuiflora* and Glycophytes

**FIGURE 7:**
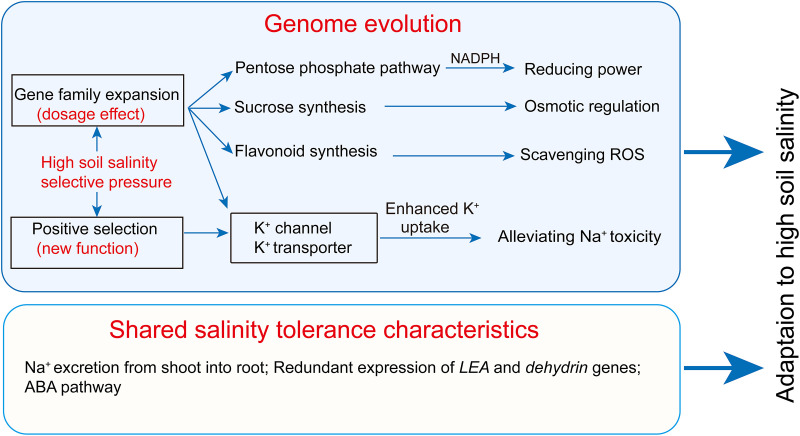
Genomic mechanisms of salinity tolerance evolution in *P. tenuiflora*. LEA, late embryogenesis abundant protein; ROS, reactive oxygen species.

According to the phylogenetic tree constructed in this study, of the 16 species included in the analysis *D. glomerata* was most closely related to *P. tenuiflora* (Figure 1). To our knowledge, *D. glomerata* is the closest sequenced species to *P. tenuiflora*. Thus, *D. glomerata* is an ideal plant species for use in comparative genomics and transcriptome analyses of *P. tenuiflora* and glycophytes. In this study, we compared the transcriptional responses of *D. glomerata* and *P. tenuiflora* to salinity stress. ABF is a central transcriptional factor for ABA signaling, *NCED* is critical gene for ABA biosynthesis, and the expression response of the *LEA* and *dehydrin* genes under salinity stress is ABA-dependent (Hauser et al., 2011; Pedrosa et al., 2015; Sah et al., 2016). Our results showed that *P. tenuiflora* and *D. glomerata* both showed enhanced expression of *ABF*, *NCED*, *dehydrin*, and *LEA* genes under salinity stress (Figure 6B). The redundant expression of a number of *dehydrin* and *LEA* genes under salinity stress is also a common salinity tolerance characteristic for *P. tenuiflora* and *D. glomerata* (Figures 6, 7). These similar gene expression responses suggested that the ABA signaling system may be conserved in *P. tenuiflora* and glycophytes. LEA and dehydrin proteins played critical roles in osmotic adjustment and the prevention of protein aggregation under salinity or drought stress (Goyal et al., 2005). Although the response pattern of the *P. tenuiflora LEA* superfamily to salinity stress was similar to that of the glycophytes (Goyal et al., 2005; Rorat, 2006), the number of *LEA5* genes in *P. tenuiflora* was higher than that in *O. sativa*, *H. vulgare*, *A. tauschii*, *B. distachyon*, and *D. glomerata*. The high number of *LEA5* subfamily in *P. tenuiflora* may contribute to its strong tolerance to salinity stress and drought because the osmotic role of LEA relies on its protein abundance (i.e., there is a dosage effect). Interestingly, the number of *SMP*, *LEA1*, and *LEA4* subfamilies in *P. tenuiflora* was much lower than that in *D. glomerata* (Figure 4A). We propose that different members or subfamilies of the *LEA* superfamily played various roles in salinity tolerance differentiation between *P. tenuiflora* and *D. glomerata*. How the *LEA* superfamily mediates salinity tolerance in *P. tenuiflora* should be further investigated in the future.

### K^+^/Na^+^ Homeostasis Mechanisms of *P. tenuiflora* Are Distinct From Those of Glycophytes

Under salinity stress, the competition between Na^+^ and K^+^ for binding sites on transporters, channels, or proteins decreases the accumulation of K^+^ in plants. Similarly, a high cytosolic K^+^/Na^+^ ratio can decrease the binding frequency of Na^+^ to enzymes or proteins with K^+^-binding sites. Therefore, high cytoplasmic K^+^/Na^+^ ratios are salinity tolerance characteristics shared by all glycophytes and some halophytes (Zhu, 2003; Ashraf, 2004). Salinity stress increases Na^+^ concentration and decreases K^+^ concentration in almost all plant species (Khan et al., 2000; Zhu, 2003; Parida and Das, 2005; Flowers and Colmer, 2008; Munns and Tester, 2008; Wang et al., 2009; Zhao et al., 2017). In the present work, root K^+^ concentration was elevated in *P. tenuiflora* under salinity stress, but it was strongly reduced in all of the tested glycophytic Gramineae plants (Figure 5B). *P. tenuiflora* may have evolved a special K^+^ uptake system that enables the roots to tolerate high external Na^+^ concentrations. In *P. tenuiflora* roots, the enhanced K^+^ concentration increased the K^+^/Na^+^ ratio and relieved Na^+^ damage to cells under salinity stress. Our comparative genomics analysis provided an explanation for the K^+^ uptake characteristics of *P. tenuiflora* under salinity stress. Some *P. tenuiflora* gene families mediating K^+^ uptake expanded during evolutionary history (Figure 2C and Supplementary Table 1). We propose that these expanded K^+^ uptake gene families may have contributed to the development of strong K^+^ uptake capacity in *P. tenuiflora* under salinity stress because the functions of K^+^ uptake genes rely on their protein abundance (dosage effect) (Figure 7). Indeed, some K^+^ channel or transporter genes were upregulated in roots of *P. tenuiflora* under salinity stress (Supplementary Table 5). Interestingly, several *P. tenuiflora* K^+^ channel genes were positively selected during evolutionary history (Table 1 and Supplementary Table 3). We propose that the selection pressure imparted by high soil salinity may have driven the *P. tenuiflora* K^+^ channel genes to evolve new functions that differ to those in glycophytes. This change may also have contributed to the development of the K^+^ uptake characteristic of *P. tenuiflora* under salinity stress. Besides relieving Na^+^ toxicity, improving the K-nutritional status also can greatly alleviate damage caused by low temperatures, iron toxicity, and drought (Cakmak, 2005). The evolution of *P. tenuiflora* K^+^ uptake genes therefore also have benefitted these plants in adapting to these other types of environmental stress.

*Puccinellia tenuiflora* exhibited an extremely low Na^+^ concentration in the salinity-stressed shoots (Figure 5A). When the seedlings were exposed to 300 mM NaCl for 48 h, the shoot Na^+^ concentration of *P. tenuiflora* was only 10.7–19.8% of that in glycophytic Gramineae plants (Figure 5A). This result was consistent with that of Wang et al. (2009) who showed that *P. tenuiflora* also exhibited extremely lower shoot Na^+^ concentrations than those of wheat. It has been reported that *P. tenuiflora* has a much stronger capacity to restrict the unidirectional Na^+^ influx from the rhizosphere into the root than glycophytes (Wang et al., 2009). Our results provided an additional insight into the Na^+^ tolerance of *P. tenuiflora*. *P. tenuiflora* may have an extremely strong capacity to control Na^+^ influx from root into shoot through Na^+^ excretion from shoot into the root and/or the restriction of unidirectional Na^+^ influx into the shoot. Na^+^ excretion from the shoot into the root is a common salinity tolerance characteristic for glycophytes (Munns and Tester, 2008; Wang and Xia, 2018; Ganie et al., 2019). Although the transporter or channel gene mediating Na^+^ excretion has not yet been identified in *P. tenuiflora*, the Na^+^ excretion channel or transporter in this species was likely to be more permeable to Na^+^ and/or incur a lower energy cost to that in other species. This hypothesis required verification using structural biology and electrophysiological techniques. In addition, the number of *P. tenuiflora HKT* and *NHX* families was higher than that of its relative *D. glomerata* (Supplementary Table 7). *HKT1;3* was upregulated in the roots of *P. tenuiflora* at 48 h under salinity stress, but no *HKT* gene was upregulated in the roots of *D. glomerata* (Figure 6B). The *HKT1* family has been demonstrated to mediate Na^+^ excretion from shoots to roots in rice, Arabidopsis, and wheat (Munns and Tester, 2008; Wang and Xia, 2018; Ganie et al., 2019). Therefore, the *P. tenuiflora HKT1;3* gene (PtO_1129.21) may be a candidate Na^+^ excretion gene, which should be investigated in the future.

### Genome Evolution Appears to Contribute Development of Salinity Tolerance in *P. tenuiflora*

Halophytes comprise remarkable plant species that can survive extremely high levels of soil salinity that would kill 99% of other plant species (glycophytes) (Flowers and Yeo, 1995; Flowers et al., 2010). It is of interest, therefore, to determine when and how halophytes achieved strong salinity tolerance and what mechanisms drive salinity tolerance differences between halophytes and glycophytes. *P. tenuiflora* may have inhabited saline environments for several millions of years and so may have evolved unique metabolic traits in response to its long-term exposure to severe soil salinity and drought. Our comparative genomics analysis revealed that the *P. tenuiflora* genes that were positively selected during evolutionary history were enriched in response to abiotic stimuli and response to stress (Figure 3). The selective pressure incurred by high soil salinity levels may have driven *P. tenuiflora* stress response genes to evolve new functions that differed from those of glycophytes, which may have accelerated the differentiation in salinity tolerance between *P. tenuiflora* and glycophytic Gramineae plants (Figure 7). In addition, expanded *P. tenuiflora* genes were found to be enriched in the PPP, flavonoid biosynthesis, and sucrose biosynthesis (Figure 2B and Supplementary Table 2). Accordingly, a *glucose-6-phosphate dehydrogenase* gene (a rate-controlling gene in the PPP), two *flavonoid 3*′,*5*′*-hydroxylase* genes (critical genes for flavonoid biosynthesis), and three *sucrose synthase* genes were upregulated in the roots or leaves of *P. tenuiflora* under salinity stress (Supplementary Table 5). The PPP is important when plants are exposed to stress because some signals trigger the rapid diversion of NADPH to use in stress responses (Kruger and Schaewen, 2003; Esposito, 2016). The enhanced PPP might provide additional energy and carbon resources to fuel salinity stress responses in *P. tenuiflora* (Figure 7). Flavonoid plays important roles in the detoxification of reactive oxygen species under salinity stress (Agati et al., 2012), and sucrose is a central compatible solute for osmotic regulation (Parida and Das, 2005; Xiao et al., 2020). Therefore, the expansion and enhanced expression of flavonoid biosynthesis and sucrose biosynthesis genes in *P. tenuiflora* protected these plants from the toxicity associated with reactive oxygen species and Na^+^ under salinity stress (Figure 7). In addition, gene families involved in the plant-pathogen interaction pathway were expended during the evolutionary history of *P. tenuiflora* (Figure 2B), which may have contributed to the development of biotic stress tolerance of *P. tenuiflora*.

In conclusion, the strong salinity tolerance of *P. tenuiflora* was linked to its K^+^ uptake characteristics and strong capacity to control Na^+^ influx into shoots, and some *P. tenuiflora* K^+^ uptake gene families experienced expansion and positive selection during evolutionary history. During the adaptation of *P. tenuiflora* to high soil salinity, expanded salinity-tolerant genes have exerted dosage effects, and positively-selected salinity-tolerant genes may have been involved in the construction of new salinity tolerance pathways. This was the genomic basis for salinity tolerance differentiation between *P. tenuiflora* and glycophytic Gramineae plants (Figure 7).

## Data Availability Statement

The original contributions generated for this study are publicly available. This data can be found here: All raw data of transcriptional analysis are available at NCBI (accession number PRJNA673532/SRR12957599-SRR12957646). The genome sequence of *P. tenuiflora* is available from NCBI (https://www. ncbi.nlm.nih.gov/genome/?term=QRDG00000000.1) (accession number QRDG00000000.1).

## Author Contributions

CY and RG: experiment design. RG, LZ, KZ, HL, NB, and CY: performance of experiments. RG, CY, LZ, and KZ: data analysis. CY, RG, LZ, and KZ: manuscript writing. All authors read and approved the final manuscript.

## Conflict of Interest

KZ was employed by Beijing Novogene Bioinformatics Technology Ltd. The remaining authors declare that the research was conducted in the absence of any commercial or financial relationships that could be construed as a potential conflict of interest.

## Footnotes

1 http://www.efloras.org/florataxon.aspx?flora_id=2&taxon_id=200026128

2 https://orthomcl.org/orthomcl/app

3 http://www.timetree.org

## Supplementary Material

Supplementary Figure 1 Gene number in *Puccinellia tenuiflora* and several sequenced Gramineae plants.

Supplementary Figure 2 Motif component comparison of *P. tenuiflora*, *Dactylis glomerata*, rice, *Hordeum vulgare*, and *Aegilops tauschii* for each NHX protein.

Supplementary Figure 3 Motif component comparison of *P. tenuiflora*, *D. glomerata*, rice, *H. vulgare*, and *A. tauschii* for each HKT protein.

Supplementary Figure 4 Motif component comparison of *P. tenuiflora*, *D. glomerata*, rice, *H. vulgare*, and *A. tauschii* for each AKT protein.

Supplementary Figure 5 Effects of salinity stress on the Na^+^/K^+^ ratio in *P. tenuiflora*, *O. sativa*, *H. vulgare*, *D. glomerata*, and *T. aestivum*. The 15-day-old seedlings were exposed to 300 mM NaCl for 12 h and 48 h. Values are expressed as means (±standard deviation, S.D.) of the three biological replicates. ^∗^ indicates significant difference between control and stress conditions within the same tissue at the 0.05 level, according to the *t*-test.

Supplementary Figure 6 KEGG enrichment of DEGs in *P. tenuiflora* under salinity stress. The 15-day-old seedlings were exposed to 300 mM NaCl for 12 and 48 h. Each treatment had three biological replicates.

Supplementary Table 1 Members of the *P. tenuiflora* gene families that expanded during evolutionary history.

Supplementary Table 2 Enriched GO terms in the GO enrichment analysis of the *P. tenuiflora* gene families that expanded during evolutionary history.

Supplementary Table 3 List of *P. tenuiflora* genes that were positively selected during evolutionary history.

Supplementary Table 4 Differentially expressed genes in *P. tenuiflora* under salinity stress. The 15-day-old seedlings were exposed to 300 mM NaCl for 12 and 48 h. Each treatment had three biological replicates.

Supplementary Table 5 Differentially expressed salinity-tolerant genes in *P. tenuiflora* under salinity stress. The 15-day-old seedlings were exposed to 300 mM NaCl for 12 and 48 h. Each treatment had three biological replicates.

Supplementary Table 6 Comparative fold changes (stress/control) of qRT-PCR and RNA-seq in *P. tenuiflora* roots at 48 h. Each treatment had three biological replicates, and each biological replicate comprised a pool of 10 plants. The gene-specific primers were displayed.

Supplementary Table 7 Number of *HKT*, *AKT*, and *NHX* families in *P. tenuiflora*, rice, *D. glomerata*, and *Arabidopsis*.

## Abbreviations

ABA: abscisic acid
NHX: Na^+^/H^+^ exchanger
PPP: pentose phosphate pathway
DEG: differentially expressed gene
NCED: 9-*cis*-epoxycarotenoid dioxygenase
ABF: abscisic acid-responsive element binding protein
HAK: KUP/HAK/KT K^+^ transporter
V-H ^+^-ATPase: vacuolar H^+^-ATPase
HKT: high-affinity K^+^ transporter
AKT: low-affinity K^+^ transporter
6PGDH: 6-phosphogluconate dehydrogenase
LEA: late embryogenesis abundant protein.
