# Supplementary figures and images for: Comparative Genomics and Transcriptomics of the Extreme Halophyte Puccinellia tenuiflora Provides Insights Into Salinity Tolerance Differentiation Between Halophytes and Glycophytes
Source: Front Plant Sci. 2021 Apr 22;12:649001. doi: 10.3389/fpls.2021.649001 (PMC8100201; doi:10.3389/fpls.2021.649001)

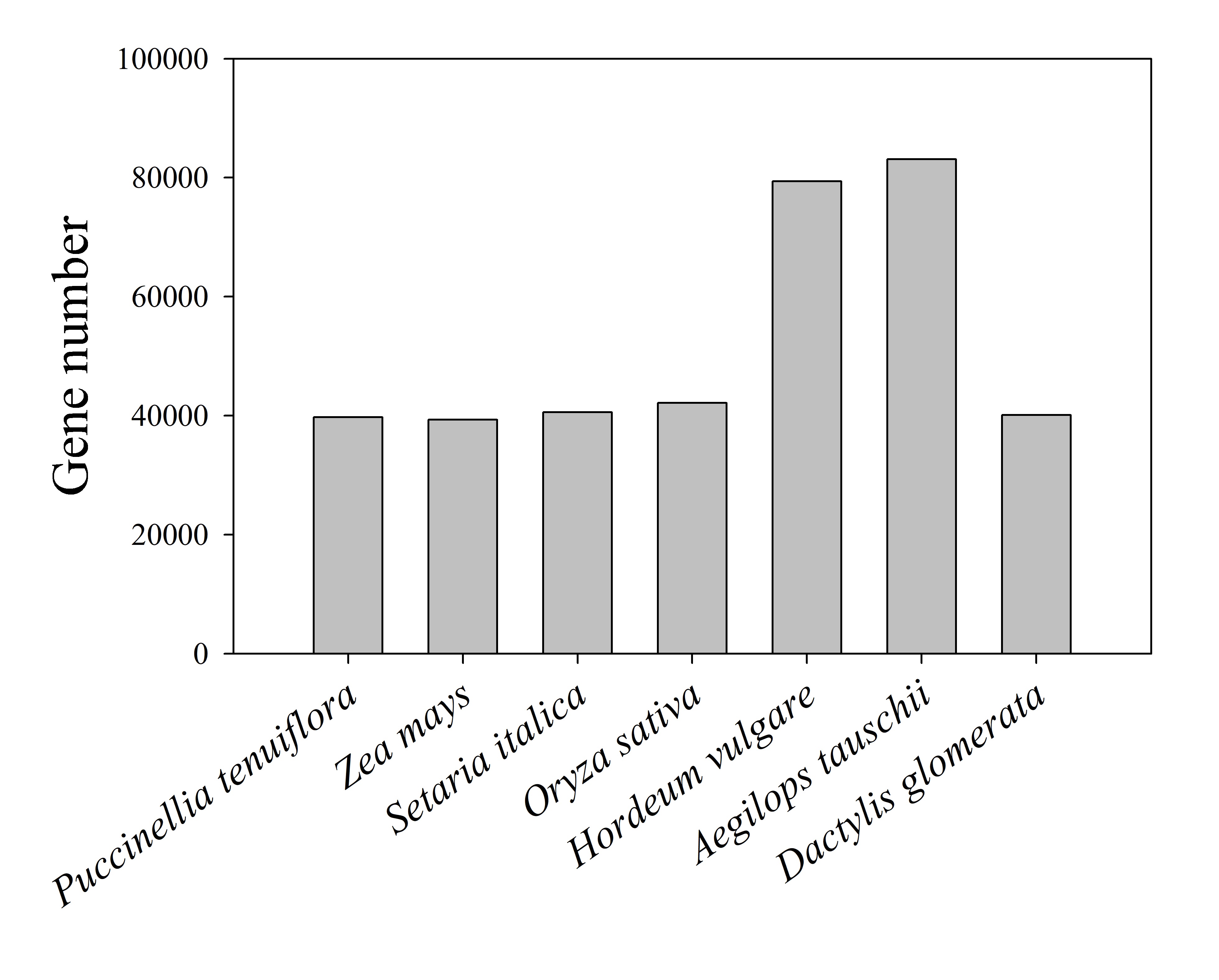

Supplement: Supplementary Figure 1 — Gene number in Puccinellia tenuiflora and several sequenced Gramineae plants. [file Image_1.JPEG]

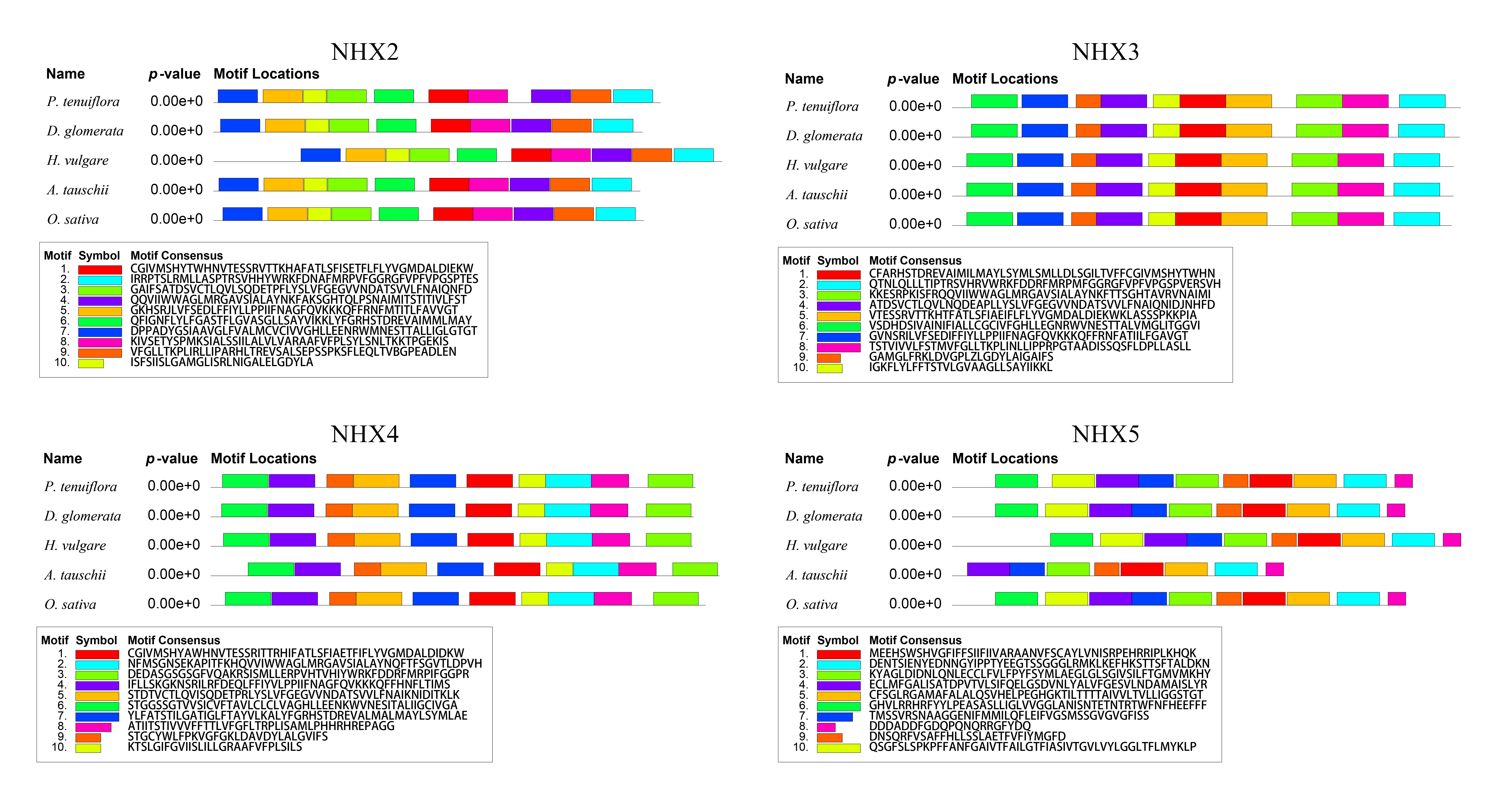

Supplement: Supplementary Figure 2 — Motif component comparison of P. tenuiflora, Dactylis glomerata, rice, Hordeum vulgare, and Aegilops tauschii for each NHX protein. [file Image_2.JPEG]

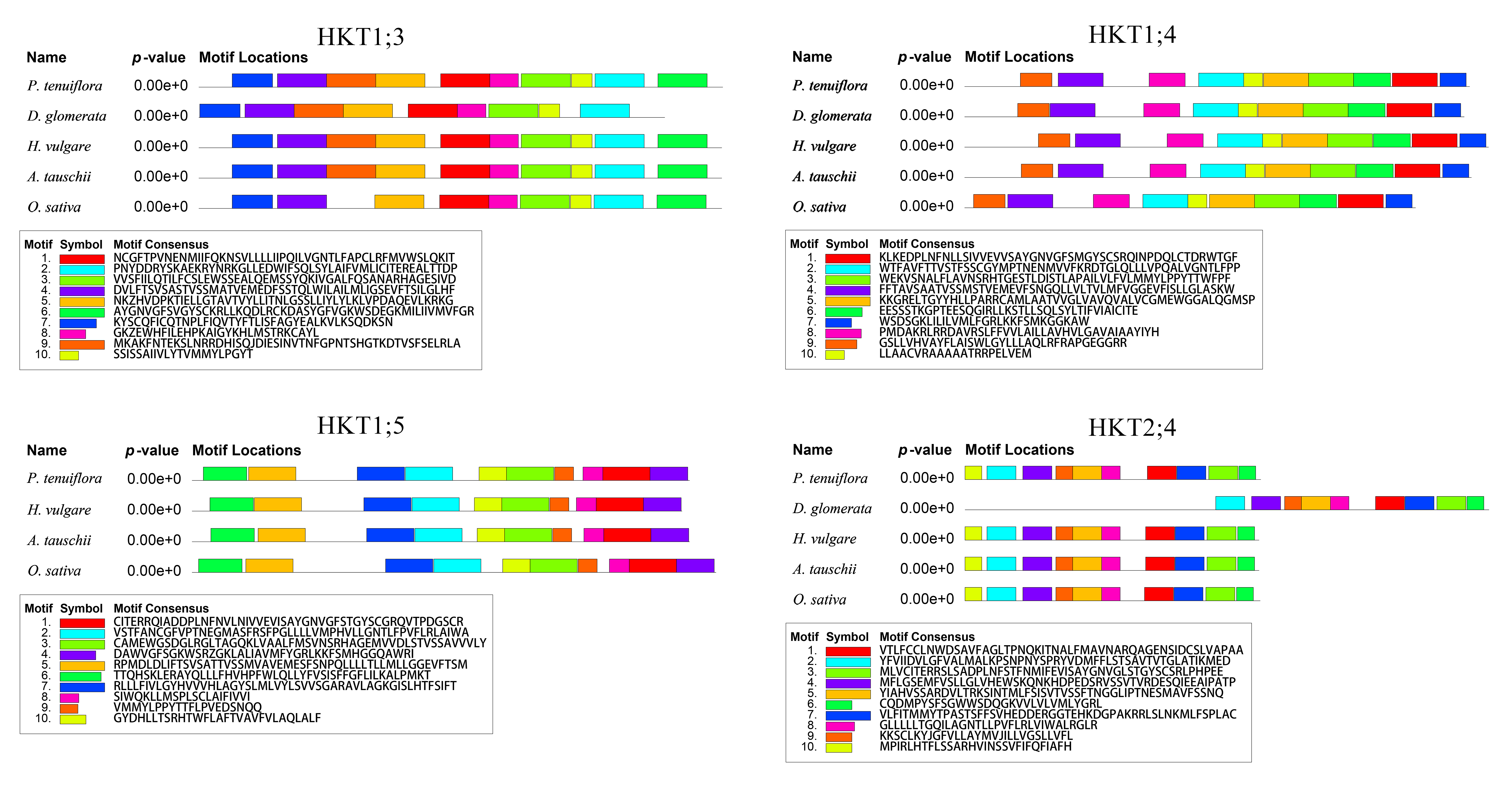

Supplement: Supplementary Figure 3 — Motif component comparison of P. tenuiflora, D. glomerata, rice, H. vulgare, and A. tauschii for each HKT protein. [file Image_3.TIF]

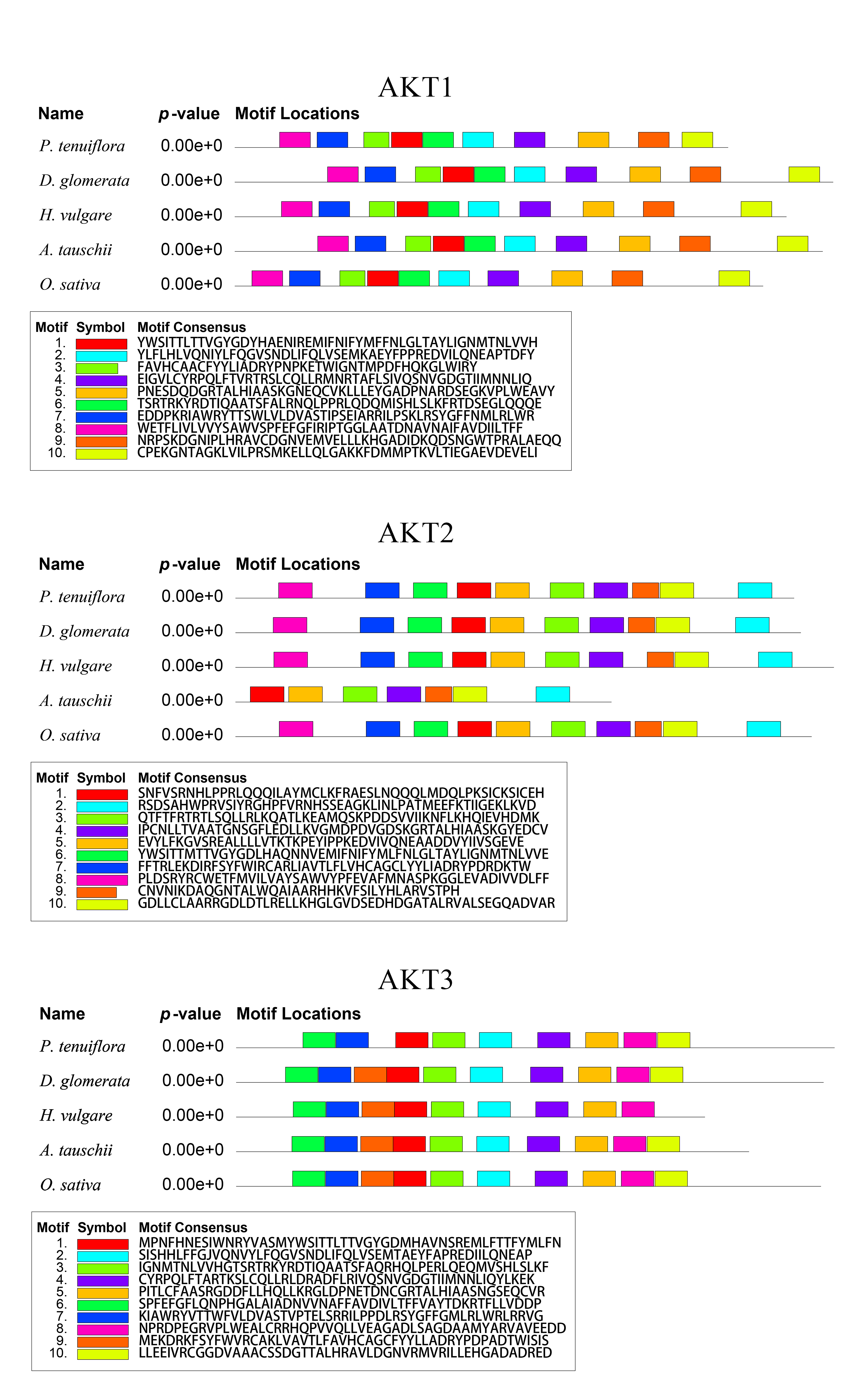

Supplement: Supplementary Figure 4 — Motif component comparison of P. tenuiflora, D. glomerata, rice, H. vulgare, and A. tauschii for each AKT protein. [file Image_4.TIF]

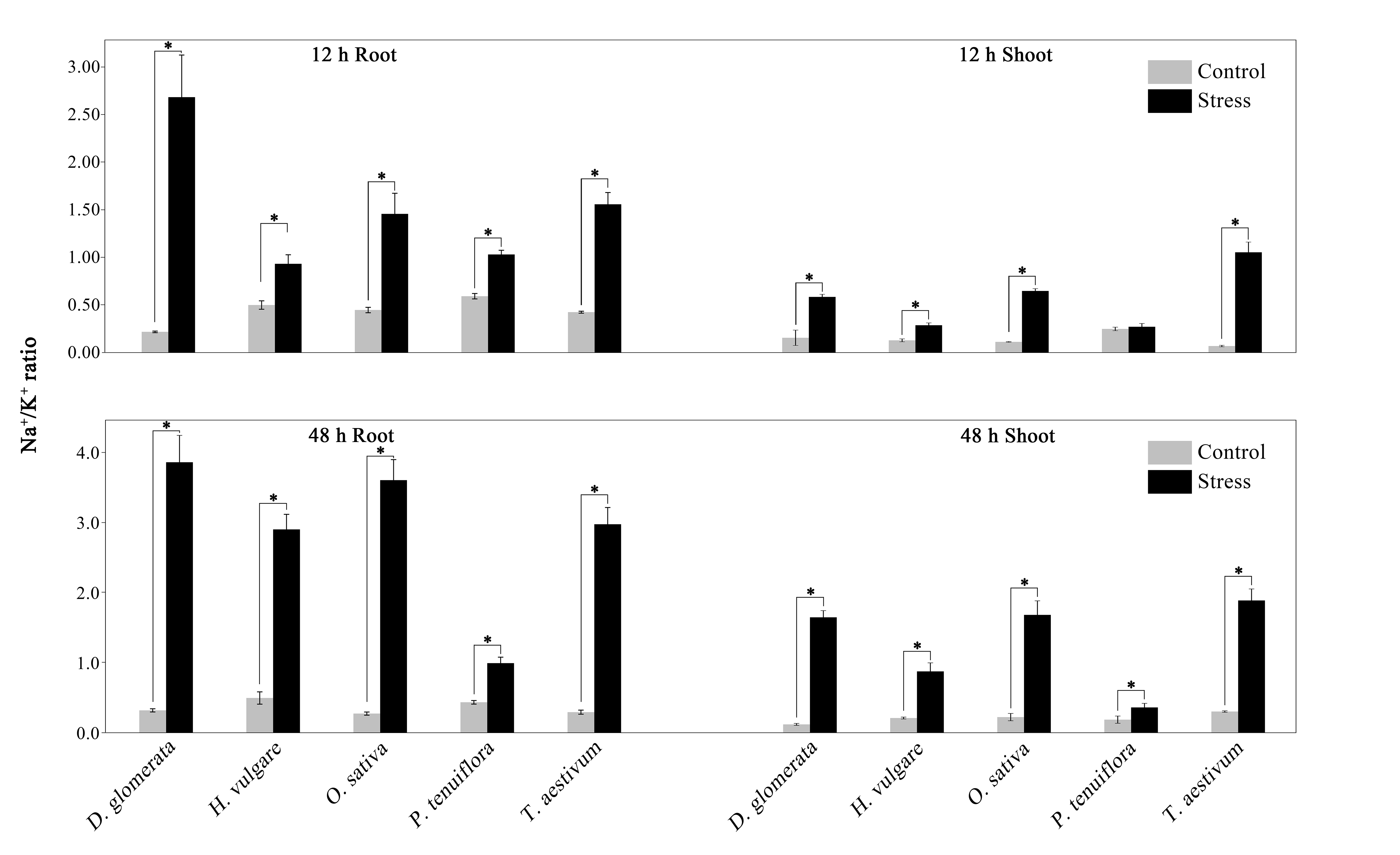

Supplement: Supplementary Figure 5 — Effects of salinity stress on the Na+/K+ ratio in P. tenuiflora, O. sativa, H. vulgare, D. glomerata, and T. aestivum. The 15-day-old seedlings were exposed to 300 mM NaCl for 12 h and 48 h. Values are expressed as means (±standard deviation, S.D.) of the three biological replicates. ∗ indicates significant difference between control and stress conditions within the same tissue at the 0.05 level, according to the t-test. [file Image_5.TIF]

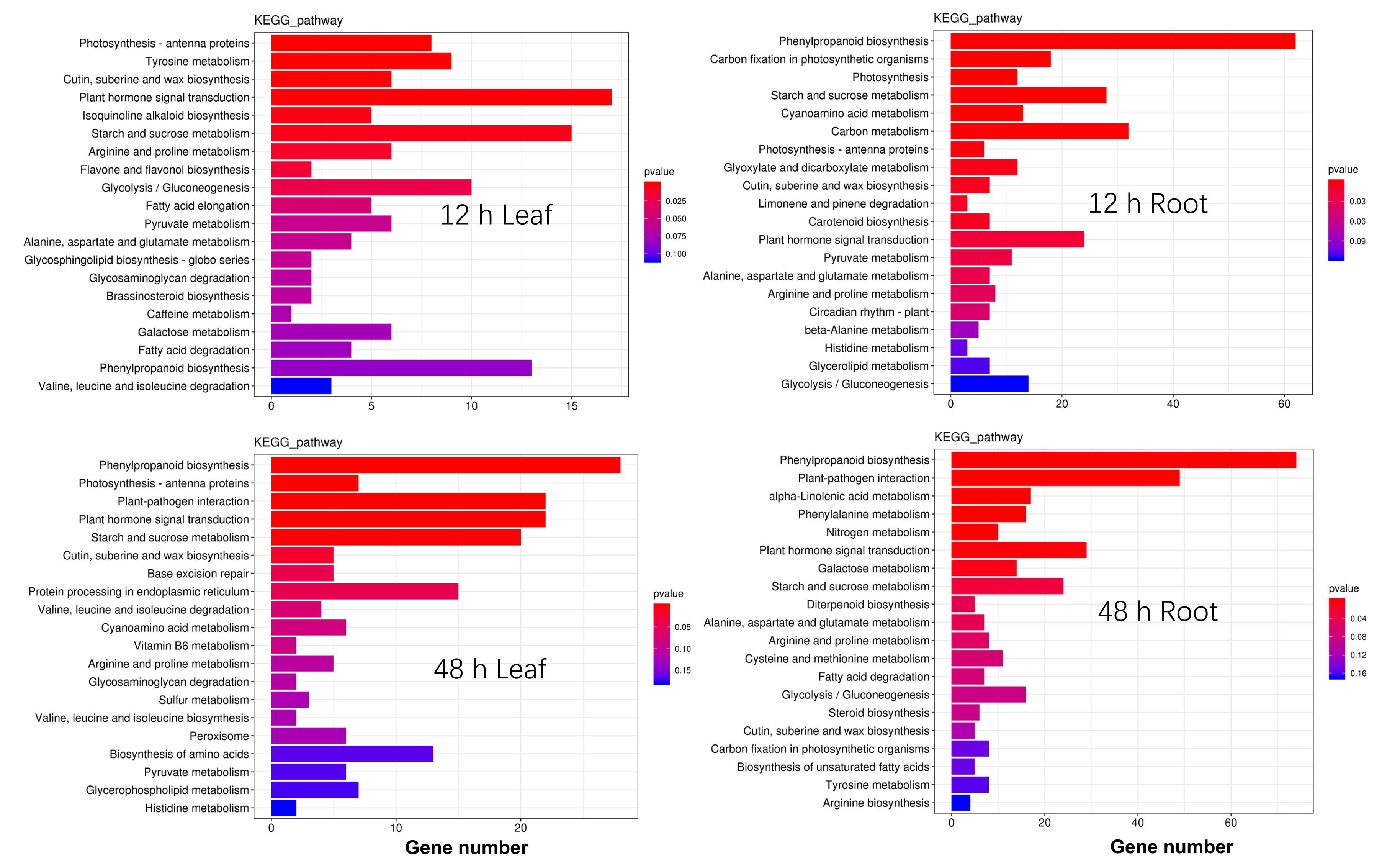

Supplement: Supplementary Figure 6 — KEGG enrichment of DEGs in P. tenuiflora under salinity stress. The 15-day-old seedlings were exposed to 300 mM NaCl for 12 and 48 h. Each treatment had three biological replicates. [file Image_6.TIF]
